# Human population, urban settlement patterns and their impact on *Plasmodium falciparum *malaria endemicity

**DOI:** 10.1186/1475-2875-7-218

**Published:** 2008-10-27

**Authors:** Andrew J Tatem, Carlos A Guerra, Caroline W Kabaria, Abdisalan M Noor, Simon I Hay

**Affiliations:** 1 Spatial Ecology and Epidemiology Group, Tinbergen Building, Department of Zoology, University of Oxford, South Parks Road, Oxford, OX1 3PS, UK; 2 Malaria Public Health and Epidemiology Group, Centre for Geographic Medicine, KEMRI – Univ. Oxford – Wellcome Trust Collaborative Programme, Kenyatta National Hospital Grounds (behind NASCOP), P.O. Box 43640-00100, Nairobi, Kenya; 3 Centre for Tropical Medicine, John Radcliffe Hospital, University of Oxford, Oxford, OX3 9DS, UK

## Abstract

**Background:**

The efficient allocation of financial resources for malaria control and the optimal distribution of appropriate interventions require accurate information on the geographic distribution of malaria risk and of the human populations it affects. Low population densities in rural areas and high population densities in urban areas can influence malaria transmission substantially. Here, the Malaria Atlas Project (MAP) global database of *Plasmodium falciparum *parasite rate (*Pf*PR) surveys, medical intelligence and contemporary population surfaces are utilized to explore these relationships and other issues involved in combining malaria risk maps with those of human population distribution in order to define populations at risk more accurately.

**Methods:**

First, an existing population surface was examined to determine if it was sufficiently detailed to be used reliably as a mask to identify areas of very low and very high population density as malaria free regions. Second, the potential of international travel and health guidelines (ITHGs) for identifying malaria free cities was examined. Third, the differences in *Pf*PR values between surveys conducted in author-defined rural and urban areas were examined. Fourth, the ability of various global urban extent maps to reliably discriminate these author-based classifications of urban and rural in the *Pf*PR database was investigated. Finally, the urban map that most accurately replicated the author-based classifications was analysed to examine the effects of urban classifications on *Pf*PR values across the entire MAP database.

**Results:**

Masks of zero population density excluded many non-zero *Pf*PR surveys, indicating that the population surface was not detailed enough to define areas of zero transmission resulting from low population densities. In contrast, the ITHGs enabled the identification and mapping of 53 malaria free urban areas within endemic countries. Comparison of *Pf*PR survey results showed significant differences between author-defined 'urban' and 'rural' designations in Africa, but not for the remainder of the malaria endemic world. The Global Rural Urban Mapping Project (GRUMP) urban extent mask proved most accurate for mapping these author-defined rural and urban locations, and further sub-divisions of urban extents into urban and peri-urban classes enabled the effects of high population densities on malaria transmission to be mapped and quantified.

**Conclusion:**

The availability of detailed, contemporary census and urban extent data for the construction of coherent and accurate global spatial population databases is often poor. These known sources of uncertainty in population surfaces and urban maps have the potential to be incorporated into future malaria burden estimates. Currently, insufficient spatial information exists globally to identify areas accurately where population density is low enough to impact upon transmission. Medical intelligence does however exist to reliably identify malaria free cities. Moreover, in Africa, urban areas that have a significant effect on malaria transmission can be mapped.

## Background

The public health burden posed by malaria has put its control high on the international development agenda. Recent improvements in the levels of funding for malaria control and their disbursement have resulted in an increase in use of insecticide treated nets and access to effective antimalarial drugs in many malaria endemic countries that are starting to have demonstrable public health impact [1-8]. The rapidly changing malaria landscape (epidemiological and political) requires an accurate and contemporary description of risk with which to help audit future needs objectively and guide resource allocations effectively and equitably [9].

The extremes of both low and high population density modify malaria transmission and have profound consequences for estimates of its public health burden [10-14]. In areas of exceptionally low population density, there may be insufficient numbers of people to support transmission [10], while urban areas cause marked entomological, parasitological and behavioural effects that result in reduced risks [13]. These effects need to be assessed to help estimate their impact on the risks of *Plasmodium falciparum *malaria globally. Recent studies have examined the potential of global population and urban extent surfaces for mapping the risks of malaria and identifying populations at risk. Guerra *et al *[15,16] estimated the global extent of malaria transmission for 2005 by using a set of rules, including the exclusion of i) all areas where population density was less than one person per km^2^; ii) urban extents of cities identified as malaria free in travel guidelines; and iii) urban extents of cities with populations greater than one million. Further, the effects of urbanization (measured by population density-based classifications of urban extents) on *P. falciparum *entomological inoculation rate (EIR) were also used to reduce endemicity in urban areas by stepwise reductions in categorized endemicity classes, before estimating populations at risk and the mortality attributable to *P. falciparum *malaria in Africa [13]. A similar logic was used to downgrade endemicity classes for the hypothesized effect of urbanization in recent global estimates of the *P. falciparum *morbidity burden [17]. Finally, population counts and a compilation of medical intelligence on malaria risk in large cities were used to define the limits of unstable *P. falciparum *malaria transmission globally [18].

This paper documents efforts to use a global population database, medical intelligence and urban extent maps to identify and describe spatially those areas of the malaria endemic world where human population densities are lowest and highest, resulting in modified transmission and, thus, impact upon malaria burden. Further, the Malaria Atlas Project (MAP) global *P. falciparum *Parasite Rate (*Pf*PR) survey database is used to explore relationships between human population density, settlement patterns and *P. falciparum *malaria transmission, in order to refine rules for data exclusion and transmission reduction required in estimating populations at risk for future mapping initiatives and burden estimates. Finally, the problems and drawbacks that exist in using global spatial population databases and counterpart urban maps as demographic components for malaria risk mapping are highlighted.

## Methods

### Datasets

#### The MAP PfPR database

**Figure 1:**
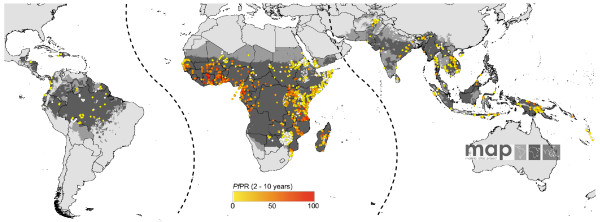
**The spatial limit of *Plasmodium falciparum *malaria risk defined by *P. falciparum *annual parasite incidence (*Pf*API) with temperature and aridity masks**[**18**]. Areas were defined as stable (dark grey areas, where PfAPI ≥ 0.1‰ pa), unstable (middle grey areas, where PfAPI < 0.1‰ pa) or no risk (light grey) [17-19]. The borders of the 87 countries defined as *P. falciparum *endemic are shown. The community surveys of *P. falciparum *prevalence conducted between 1985 and 01 March 2008 are plotted. Of the 4,887 surveys that could be geo-positioned, 4,077 fell within the predicted limits of *P. falciparum *malaria risk. The data shown are age-standardized (*Pf*PR_2–10_) and shown as a continuum from 0–100%. The dashed lines indicate the separation between the area defined as 'Africa+' (Africa, Yemen and Saudi Arabia) and the rest of the world used in the analyses.

The rigorous process of identifying, assembling and geo-locating community-based survey estimates of *P. falciparum *parasite prevalence undertaken since 1985 has been described previously [19,20], where *Pf*PR estimates have been reported in a wide variety of age groupings. To standardize to a single, epidemiologically important age range (2.00–9.99 years), an algorithm based on catalytic conversion models first adapted for malaria by Pull and Grab [21] and described in detail elsewhere [22] was applied. Surveys that could only be geo-referenced to administrative unit polygons, rather than precise locations, were removed before analysis to ensure that only the most accurately geo-located surveys were used [23]. A total of 3,525 geo-referenced survey points were used in the analyses, of which, 2,376 (903 rural, 292 urban, 1,144 unclassified) were in Africa, 109 (97 rural, two urban, seven unclassified) in the Americas and 1,040 (428 rural, 15 urban, 506 unclassified) in the central and south-east Asia region. Figure 1 shows the distribution of *Pf*PR surveys used in this study overlaid on the spatial limits of malaria transmission [18], with dividing lines showing the 'Africa+' region (Africa, Yemen and Saudi Arabia). This was analysed separately from the rest of the world in this paper.

#### Global population databases

The principal source of human population distribution data used for assessing the effects of low and high population densities on *Pf*PRs in this study was the Global Rural Urban Mapping Project (GRUMP). The GRUMP spatial population database provides global gridded population density estimates at ~1 km spatial resolution. The data and methods used to construct GRUMP are described elsewhere [24,25]. In brief, the most recent census, and other demographic data at the highest possible administrative boundary level available, were obtained for every country in the World, and areal weighted [26]. An urban extent mask, GRUMP-UE (described below), was then used to adjust population numbers within each extent to match estimated totals for each settlement in question. Finally, the individual national population surfaces were projected to the years 1990, 1995 and 2000 and adjusted to match the national population totals estimated by the United Nations' Population Division [27]. The clearly documented methods, the substantially larger number of administrative units used to create the database, and tests showing its higher accuracy over other products [26,28], have led to the use of GRUMP for the current analyses.

**Figure 2:**
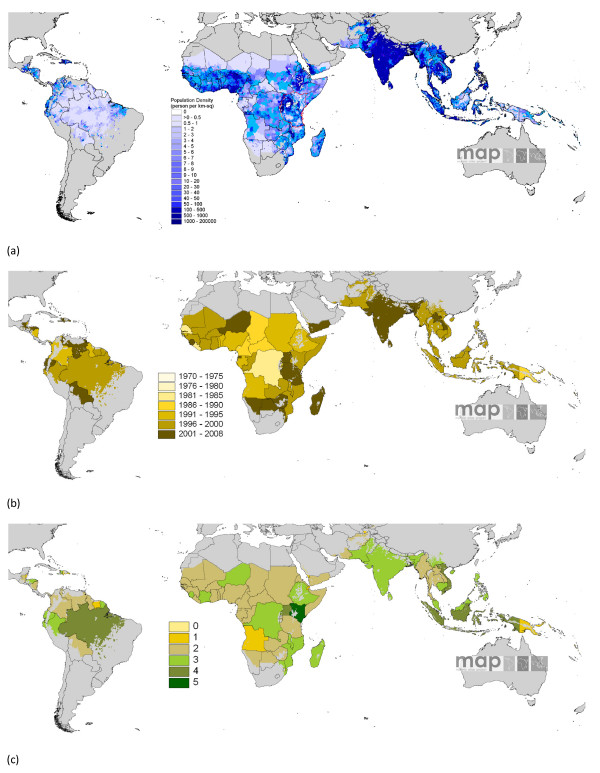
**(a) Modified GRUMP **[**24**]** masked by the limits of***** Pf *****malaria transmission **[**18**]** and projected to 2007 **[**27**]. The red border highlights where data from Tatem *et al *[28] were inserted. (b) The date of the census data used in the construction of the population surface in (a), and (c) the administrative unit level of census data used in the construction of the population surface in (a).

UN-adjusted population counts and densities for the year 2000 were obtained from [27]. For Burundi, Kenya, Rwanda, Tanzania and Uganda, the mapped surfaces were replaced with those described in Tatem *et al. *[28], as they have been shown to be more accurate. Prior to replacement the surfaces were adjusted to 2000 and degraded to the same approximate 1 km spatial resolution as GRUMP. The entire global surface was projected to 2007 by applying national, medium variant, inter-censal growth rates by country [27], using methods previously described [26]. Figure 2 shows the resultant population density surface, alongside the year and administrative boundary unit level of the census data used to make the surface. Each is masked by the limits of malaria transmission [18], as defined in Figure 1.

The use of an urban extent mask to adjust population totals and densities within areas defined as urban, means that GRUMP estimates of urban population densities are biased by the specific features of this mask. Moreover, the mask has been shown to overestimate consistently the extent of urban areas [29,30]. Given the aim of this study to also examine the effects of population densities on *Pf*PRs *within *urban areas, subsequent to the choice of the most accurate urban map, a population database unbiased by choice of urban definition was required. For this, the Gridded Population of the World version 3 (GPW3) [24] population density database was obtained and projected to 2007. GPW3 uses the same areal weighted census data as GRUMP as input, but implements no urban adjustments and therefore, unlike GRUMP, is independent of urban definition, making it useful for examining population density variations within urban extents that have been mapped independently.

#### Global urban maps

To assess the ability of existing datasets to identify areas where urbanization has a significant effect on malaria transmission, a set of public domain global urban maps was obtained (Table 1) [13,15,27,31-36]. The ambiguity over what constitutes an urban area and consequently, urbanization, has led to several attempts to generate global and continental-scale urban maps using consistent methods, and many of these were analysed in Tatem *et al *[29]. For this study, the maps documented in Table 1, which include the 'urban' classes from two global satellite sensor-derived land cover maps (Advanced Very High Resolution Radiometer, AVHRR, and Moderate Resolution Imaging Spectroradiometer, MODIS) and the 'populated built up area' features from military mapping data (VMAP0), were acquired. The full GRUMP-UE map, created principally using night-time lights satellite imagery [24,37], was also acquired, as was an edited version of GRUMP-UE containing only the extents of those 123 cities within the limits of malaria transmission with populations greater than 750,000, as defined by the United Nations World Urbanization Prospects 2005 database [38] (Edited GRUMP-UE). Finally, population density based urban extent maps were created following the rules outlined in Guerra *et al *[15] and Hay *et al *[13]. In each case more contemporary population projections [27] and urban population totals [38] were used. Moreover, the Hay *et al *methodology was applied to both GRUMP and GPW3, with the 'urban' and 'peri-urban' (areas around urban extents with substantially higher population densities than the surrounding rural areas) classes merged into a single urban class, alongside also maintaining the 'urban' class and assigning the remaining classes to 'rural'.

**Table 1:** Features of each of the six global urban maps tested in this study

**Urban Map**	**Spatial Resolution**	**Production Year**	**Reference/Source/Data used**
AVHRR	1 km	1998	[31]
VMAP0	Vector polygons: 1:1000000	1997	[32]
GRUMP-UE	1 km	2004	[33,34]
Edited GRUMP-UE	1 km	2008	[27,33,34]
GUERRA	1 km	2008	[15]
MODIS	1 km	2002	[35,36]
HAY-GPW-U	5 km	2008	[13]
HAY-GPW-U-PU	5 km	2008	[13]
HAY-GRUMP-U	1 km	2008	[13]
HAY-GRUMP-U-PU	1 km	2008	[13]

GRUMP-UE = Global Rural-Urban Mapping Project Urban Extents; AVHRR = AVHRR Global Land Cover Classification urban land cover class; MODIS = MODIS Land Cover Product Binary Data from Boston University; VMAP0 = Vector map level zero; GUERRA = Population density based map described in Guerra *et al*. [15]; HAY-GPW-U = Population density based map described in Hay *et al. *[13], maintaining 'urban' class as urban and the remaining 3 classes merged to form the rural class; HAY-GPW-U-PU = Same as previous map, but merging 'urban' and 'peri-urban' classes into a single urban class; HAY-GRUMP-U = Same as previous map, but classes were defined using GRUMP and maintaining 'urban' class as urban and the remaining 3 classes merged to form the rural class; HAY-GRUMP-U-PU = same as previous map, but merging 'urban' and 'peri-urban' classes into a single urban class.

#### International travel and health guidelines (ITHGs)

Some urban areas within malaria endemic regions are known to be malaria free, and thus can be eliminated from further analyses. Information on such areas are provided by ITHGs, the two most comprehensive of which, the 'Health Information for International Travel' produced by the Centers for Disease Control [39] and the 'World malaria risk chart' produced by the International Association for Medical Assistance to Travellers [40], were obtained. These were then cross-referenced to identify any urban areas within malaria endemic countries identified explicitly as malaria free.

#### Satellite vegetation index stratification

**Figure 3:**
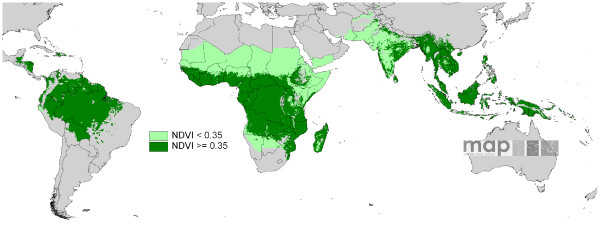
**The Normalized Difference Vegetation Index (NDVI) classes used in this study.** The NDVI was calculated from AVHRR, then classified into NDVI < 0.350, corresponding to drier, relatively less vegetated areas and NDVI > = 0.35, corresponding to wetter, relatively more vegetated areas.

To provide a basic division between high and low *Pf *transmission areas for analyses, while maintaining sufficient data, a vegetation index-based stratification was created. High and low vegetation zones were defined by the mean annual Normalized Difference Vegetation Index (NDVI) [41], as measured by the AVHRR satellite sensor. This imagery is described in detail elsewhere [42]. The NDVI image was split into two classes that have proven to be relevant to malaria ecology [13,43,44], whilst ensuring that sufficient numbers of surveys remained in each class for statistical tests. These classes were, class 1: NDVI < 0.35 (1036 surveys), corresponding to drier, less vegetated areas and class 2: NDVI > = 0.35 (2489 surveys), corresponding to wetter, more vegetated areas (Figure 3). For each of the following tests, analyses were performed globally and then separated between Africa+, and the remainder of the world. This division allowed the biogeographically, entomologically and epidemiologically distinct regions [45,46] to be considered separately, whilst retaining sufficient data in each region for meaningful analysis.

### Analyses

#### Identifying low population densities

To assess the effects of applying different thresholds of low population density on land area, population numbers and *Pf*PR surveys excluded, the modified GRUMP surface was reclassified to highlight those areas where population density was = 0, < = 0.01, < = 0.02.... < = 0.9, < = 1 persons per km^2^. For each classification, the total area covered by the class in question was calculated, as well as the total population numbers and details of *Pf*PR surveys included. These data were plotted to visualise the effects of the different low population density thresholds. For each threshold, the mean *Pf*PR of the surveys excluded was compared to those remaining using a Mann-Whitney U-test [47], to determine if such thresholds identified surveys with significantly lower *Pf*PRs. Additionally, the effects of using different categories of areas gazetted as national parks, reserves, etc, to identify low population densities were investigated, and are described in additional file 1: Gazetted Areas.

#### High population densities and urbanization

**Figure 4:**
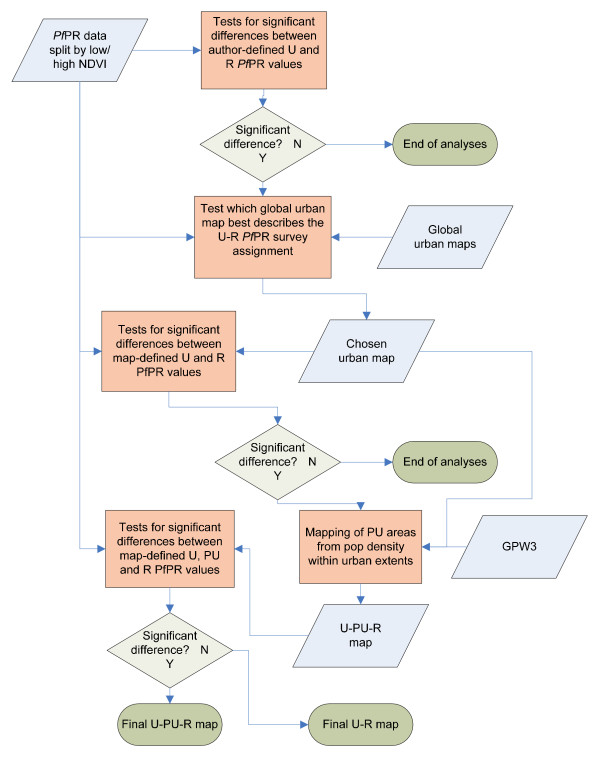
**Steps taken in the analysis of the effects of high population densities and urbanization on *Pf*PRs.** Analyses were undertaken globally and split between Africa+ and the rest of the world.

A simplified summary of the main steps taken in the analysis of the effects of high population densities and urbanization on *Pf*PR is shown in Figure 4. Firstly, the features of the *Pf*PR surveys falling within those areas defined as urban by each map detailed in Table 1 were examined to assess whether spatial information on urban extents can be used as a mask of zero transmission, as has been implemented in the past [15,16].

Secondly, author defined rural/urban classifications of *Pf*PR surveys were used to examine whether previously documented effects of urbanization on malaria transmission, based on an examination of EIR and other health metric data [13], existed for *Pf*PRs, both globally and between Africa+ and the rest of the world. Of the 3525 *Pf*PR surveys considered in this paper, 50.5% included an author-defined indicator of whether the survey was carried out in a 'rural' or 'urban' community. With the inaccuracies, inconsistencies and difficulties that exist in mapping urban extents over the malaria endemic regions of the world [29,48,49], these author-defined assignments, while resulting from partly subjective decisions and perceptions, represent the most reliable indicators available on the rural/urban setting of each *Pf*PR survey, and are made using criteria cognisant of local malaria epidemiology. To assess whether significant differences in urban and rural transmission existed, sets of proximate (spatially and temporally) urban and rural surveys were identified for comparison. For every survey identified by the survey author as being carried out within an urban area, the nearest surveys carried out in rural locations (again defined by the survey author) within 100 km and 5 years were identified. If no such surveys were found, then this urban survey was dropped from the analyses. Of the rural surveys (conducted within 100 km and 5 years of each urban survey in question) identified, the mean *Pf*PR was calculated and assigned to that urban survey to make a rural-urban pair of *Pf*PRs for analysis. These rules produced a set of urban-rural *Pf*PR survey pairs, from which tests of the difference between urban *Pf*PR values against rural values could be made. Given the skewed distribution of *Pf*PR values in the MAP database [20], the Wilcoxon signed-ranks test for paired variables [50], a nonparametric alternative to the t-test, was used to assess the differences. Tests were undertaken globally, by NDVI class and by continent (Africa+ compared with the rest of the world) to identify differing trends.

Third, if significant differences were found to exist between the author-defined urban and rural *Pf*PR values, a map which replicated these definitions accurately was required to provide a consistent, malaria-relevant basis for identifying those areas that are urbanized sufficiently to affect transmission. Moreover, the map would enable the consistent assignment of an urban/rural class to the entire set of *Pf*PR surveys, when only 50.5% of surveys currently have an author-defined classification. Again taking the author-defined urban/rural classification as being most reliable in terms of local malaria epidemiology, the ten different maps of urban extent (Table 1) were tested to examine which one described this author-based assignment most accurately. The *Pf*PR surveys with an author-defined rural/urban assignment were overlaid on each urban extent map using GIS software and the urban/rural map class was extracted for each point. These class assignments were then compared to the author assignments using confusion matrix-based statistics: percentage correct, producer's accuracy, user's accuracy and kappa [51].

Fourth, the urban map determined to be the most accurate in matching the author-defined classifications was then tested to examine the influence of urbanization on *Pf*PR using the entire set of *Pf*PR surveys with their new urban-rural assignments from the map. The *Pf*PR survey locations were overlaid on the chosen urban map and the urban/rural class assignment was extracted for each point. As described above, sets of spatially and temporally proximate urban and rural surveys were again identified to check for significant differences between urban and rural *Pf*PRs. For each point classified as urban, the surveys classified as rural within 100 km and 5 years were identified and their *Pf*PRs averaged, to create a set of map-defined urban-rural *Pf*PR pairs. This set was subject to Wilcoxon signed-rank tests to ascertain if significant differences in *Pf*PR values existed between urban and rural surveys.

Fifth, to examine local variations in urban-rural *Pf*PRs, where possible, the corresponding urban extents within the chosen urban map of the 123 UN-defined cities, as described in Table 1, were identified, and the centroids of each extent calculated. For each city, a 100 km wide circular buffer centred on the city centroid was created. Cities for which at least two *Pf*PR surveys fell within the city extent (urban) and at least two *Pf*PR surveys fell within the remainder of the 100 km buffer (rural) were identified. For these cities, the urban and rural *Pf*PR values were compared and Mann-Whitney U-tests were undertaken to assess the significance of any differences seen.

Finally, given the known difficulties in mapping urban extents that lead to extent overestimation [29,30] and the effects of 'peri-urban' areas on transmission [13], the distribution of population densities within urban areas was examined to assess whether mapped peri-urban areas had a discernible effect on *Pf*PRs, when compared to those from urban and rural areas. As described above, its independence from urban adjustment meant that GPW3 was used to map these peri-urban areas. The threshold in defining 'urban' populations, of 1000 people per km^2 ^or above, as shown to be relevant to malaria transmission by Hay *et al. *[13], was mapped within urban areas, as defined by the chosen urban map. The urban *Pf*PRs above and below this threshold were then examined to ascertain if significant differences existed. For each *Pf*PR survey within the high population density urban area ('urban'), a corresponding set of lower population density urban area surveys ('peri-urban') and rural area surveys were identified, ensuring that these two sets of surveys were undertaken within 100 km and 10 years (to ensure sufficient numbers of points for testing) of the 'urban' survey. The averages of the peri-urban and rural sets of *Pf*PR surveys were calculated and assigned to their urban *Pf*PR counterpart to make location-specific sets of urban, peri-urban and rural *Pf*PRs for testing of any trends in transmission rates by urban gradation. The nonparametric Friedman's ranking test for related samples [52] was undertaken to assess the significance of any differences.

## Results

### Low population densities

**Figure 5:**
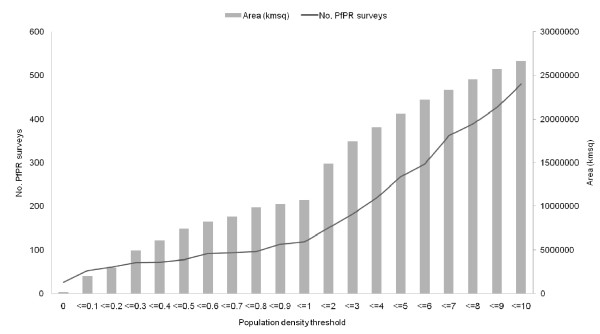
**Land area and number of *Pf*PR surveys excluded by varying population density thresholds.** GRUMP was reclassified using the thresholds on the x-axis, and for each threshold level, the land area below the threshold was calculated, as well as the number of *Pf*PR surveys within this area.

Figure 5 shows the results of low population analyses on the entire *Pf*PR survey dataset, not stratified by NDVI or continent. Figure 5 illustrates that even the most conservative threshold, identifying areas where population density is zero excludes many *Pf*PR surveys. Raising this threshold merely increases the number of *Pf*PR surveys excluded, with a consistent 80% or more of those excluded being non-zero *Pf*PR surveys. Globally, and for each NDVI class and continent, no significant differences in *Pf*PR were observed between those surveys within the low population density exclusion zones and the remainder of the surveys at any threshold.

### High population densities and urbanization

Those cities within malaria endemic countries that are identified explicitly as malaria free by the ITHGs are listed in Table 2. Table 3 shows the features of *Pf*PR surveys conducted within urban areas as defined by various criteria. In every case, over 87% of surveys conducted in areas mapped as 'urban' found *Pf*PRs greater than zero, though in many cases, only a small number of those areas mapped as urban had a survey conducted within them.

**Table 2:** Cities within malaria endemic countries that are reported by the ITHGs as being malaria free

COUNTRY	CITY	COUNTRY	CITY
Bangladesh	Dhaka	Panama	Colon
Brazil	Belem city	Panama	Panama city
Cambodia	Phnom Penh	Peru	Lima
Colombia	Barranquilla	Philippines	Manila
Colombia	Bogota	Saudi Arabia	Jiddah
Colombia	Cali	Saudi Arabia	Mecca
Colombia	Cartagena	Saudi Arabia	Medina
Colombia	Manizales	Saudi Arabia	Tai'if
Colombia	Medellin	Sri Lanka	Colombo
Ecuador	Cuenca	Thailand	Bangkok
Ecuador	Guayaquil	Thailand	Chiang Mai
Ecuador	Quito	Thailand	Songhkla
Eritrea	Asmara	Venezuela	Barinas
Ethiopia	Addis Ababa	Venezuela	Caracas
Guatemala	Guatemala city	Venezuela	Isla de Margarita
Guyana	Georgetown	Venezuela	Macuto
Guyana	New Amsterdam	Venezuela	Maracaibo
Indonesia	Denpasar	Vietnam	Da Nang
Indonesia	Jakarta	Vietnam	Haipong
Indonesia	Surabaya	Vietnam	Hanoi
Lao DPR	Vietiane	Vietnam	Ho Chi Minh
Myanmar	Magwe	Vietnam	Nha Trang
Myanmar	Mandalay	Vietnam	Qui Nhon
Myanmar	Pegu	Yemen	Sana'a
Myanmar	Sagaing	Zimbabwe	Bulawayo
Myanmar	Yangon	Zimbabwe	Harare
Nepal	Kathmandu		

**Table 3:** Features of *Pf*PR surveys mapped as urban by the various global urban maps outlined in Table 1

**Urban Map**	**No. *Pf*PR points in 'urban' areas**	**No. *Pf*PRs > 0**
AVHRR	40	39
VMAP0	35	34
GRUMP-UE	423	371
Edited GRUMP-UE	134	125
GUERRA	150	92
MODIS	127	101
HAY-GPW-U	210	194
HAY-GPW-U-PU	943	713
HAY-GRUMP-U	360	317
HAY-GRUMP-U-PU	998	827

**Figure 6:**
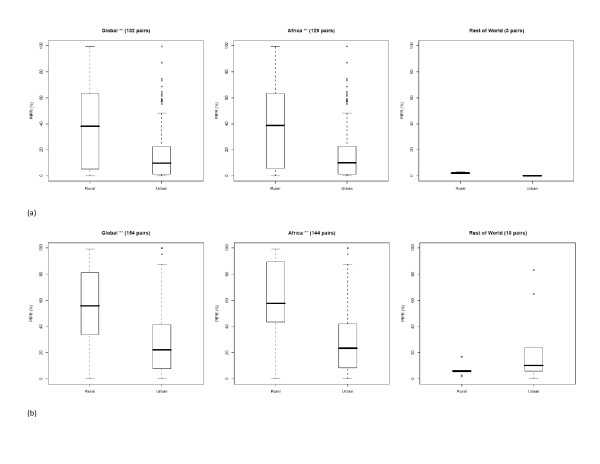
Boxplots showing the differences in *Pf*PRs by author-defined rural-urban survey pairs for surveys where mean annual NDVI is (a) less than 0.35 and (b) greater than or equal to 0.35 (* = p < 0.05, ** = p < 0.01).

The results of Wilcoxon signed-ranks tests between *Pf*PR values from the paired urban and rural sites, as defined by the survey author, are shown in Table 4. Results indicate clearly that strong significant differences exist between *Pf*PRs sampled in urban and rural locations globally, with urban areas exhibiting consistently lower values (average difference = 30.54%). This difference is due to Africa+ only, as similar results were not found for the *Pf*PR surveys across the rest of the world, with no significant differences found between rural and urban surveys, though the sample size was small. Figure 6 shows boxplots for these pairs, stratified by NDVI class, and emphasises the difference between author-defined urban and rural *Pf*PRs in Africa+, with the surveys conducted in the higher NDVI class showing a greater separation.

**Table 4:** Results of Wilcoxon signed-ranks tests on *Pf*PR values between author-defined urban

		U > R		R > U		U = R		
Dataset	No. pairs	cases	mean rank	cases	mean rank	cases	Z	Signif.
All	286	57	81.62	218	152.74	11	-10.85	**
Africa and Yemen	273	50	70.07	212	145.99	11	-11.178	**
Rest of World	13	6	4.5	7	9.14	0	-1.293	

(U) and rural (R) survey pairs (* = p < 0.05, ** = p < 0.01).

Table 5 shows the results of the accuracy assessments carried out to determine which global urban map best described the author-defined urban/rural assignments. GRUMP-UE produced the most accurate match, with the largest percentage correct and a kappa of 0.624. Following this, Table 6 shows the results of Wilcoxon signed-ranks tests between *Pf*PR values from paired urban and rural sites, as defined by GRUMP-UE. Results again show clearly that strong significant differences exist between urban and rural *Pf*PRs in Africa+, while no significant differences are found outside of Africa+, though sample sizes were small. Stratification by NDVI values shows that the urban-rural differential in *Pf*PR values is again more pronounced in the wetter, greener areas than in the drier areas, where NDVI is low. The Z values highlight, however, that the differences are not quite as clear as they are when defined by the survey author (Table 4), indicating both the difficulties in mapping urban areas globally and potential inconsistencies in author definitions of urban.

**Table 5:** Results of comparisons between the author-defined rural/urban assignment of *Pf*PR survey sites and the rural/urban assignment of the same sites by three global urban maps

		Producer's accuracy		User's accuracy		
Urban Map	Overall Percentage Correct	Rural	Urban	Rural	Urban	Kappa
AVHRR	83.62%	100%	10%	83%	100%	0.156
VMAP0	79.94%	99%	7%	80%	75%	0.093
MODIS	82.43%	92%	18%	84%	61%	0.206
GUERRA	86.74%	99%	32%	87%	88%	0.408
GRUMP-UE	89.14%	95%	65%	92%	73%	0.621
Edited GRUMP-UE	87.31%	99%	34%	87%	93%	0.439
HAY-GPW-U	87.24%	98%	35%	88%	83%	0.437
HAY-GPW-U-PU	80.29%	87%	49%	89%	45%	0.347
HAY-GRUMP-U	87.33%	95%	57%	90%	72%	0.561
HAY-GRUMP-U-PU	80.29%	87%	49%	89%	45%	0.347

The producer's accuracies show, of those defined by the authors as urban/rural, the percentage classified correctly, while the consumer's accuracies show, of those classified as urban/rural by the global map in question, the percentage of surveys classified correctly. The kappa statistic compares agreement between each map and the author-defined definitions against that which may be expected by chance, with values ranging from 1 (perfect agreement) to 0 (no different from chance agreement) to -1 (perfect disagreement).

**Table 6:** Results of Wilcoxon signed-ranks tests on *Pf*PR values between GRUMP-UE defined urban (U) and rural (R) survey pairs (* = p < 0.05, ** = p < 0.01)

		U > R		R > U		U = R		
Dataset	No. pairs	cases	mean rank	cases	mean rank	cases	Z	Signif.
All	360	85	109.09	251	188.62	24	-10.682	**
Africa and Yemen	316	69	101.12	238	169.33	9	-10.704	**
Rest of World	44	16	12.75	13	17.77	15	-0.292	
NDVI < 0.35	141	36	44.56	101	77.71	4	-6.709	**
NDVI > = 0.35	219	49	64.12	150	111.72	20	-8.369	**

**Figure 7:**
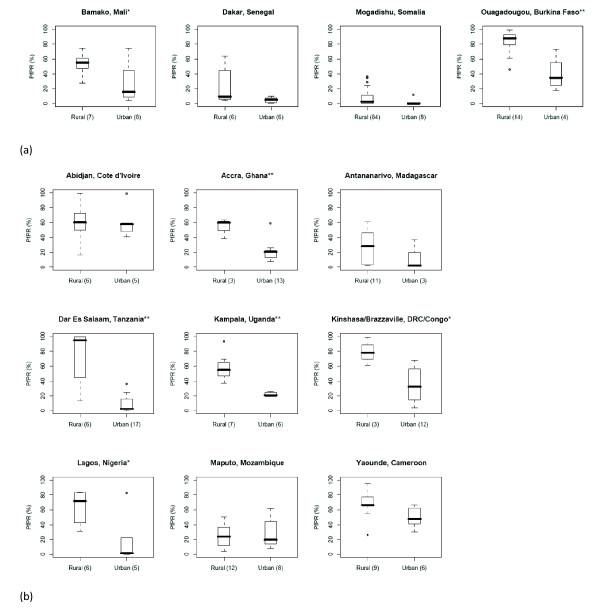
Boxplots showing the differences in *Pf*PR values by GRUMP-UE defined rural/urban assignment for cities where mean annual NDVI is (a) less than 0.35 and (b) greater than or equal to 0.35 (* = p < 0.05, ** = p < 0.01). The number of points in each class is shown in brackets below each plot.

Boxplots of *Pf*PR values by GRUMP-UE urban/rural assignments for individual African cities where more than one urban and one rural *Pf*PR survey were available are shown in Figure 7. The consistent pattern of lower *Pf*PR values in urban areas than found in the surrounding rural regions is again evident. Overall, and for many individual cities, these differences are highly significant. For those cities where the differences seen in the boxplots were not found to be significant, this is likely due to insufficient numbers of survey points to achieve significance.

**Figure 8:**
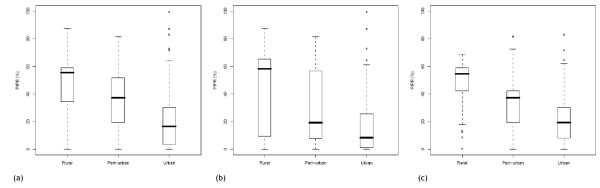
Boxplots showing the differences in *Pf*PRs by GRUMP-UE/GPW3-defined rural, peri-urban, urban survey sets for (a) all surveys in Africa+ and those where mean annual NDVI is (b) less than 0.35 and (c) greater than or equal to 0.35 (* = p < 0.05, ** = p < 0.01).

**Figure 9:**
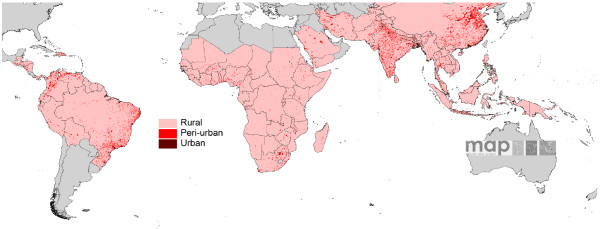
**Peri-urban and urban extents for all malaria endemic countries within the limits of *Pf *malaria transmission. **The extents are a combination of GRUMP urban extents and GPW thresholded population densities, defined using the MAP *Pf*PR database.

The urban extents in GRUMP-UE were reclassified to urban and peri-urban based on the GPW3 population density threshold, and 121 individual sets of proximate urban, peri-urban and rural *Pf*PR surveys were identified for Africa+. Figure 8 shows the boxplots of all of these combined, and stratified by NDVI class. In each case, clear differences are seen, with the Friedman test indicating that these differences are highly significant (p < 0.01). Figure 9 shows the peri-urban and urban extents mapped globally, within the limits of malaria transmission.

## Discussion

The rapid demographic shifts occurring across the developing world [53] are having profound effects on rates of morbidity and mortality attributed to many diseases and conditions [54]. These shifts impact upon malaria transmission and create a need for accurate and epidemiologically relevant spatial information on population distributions and urbanization to calibrate populations at risk (PAR) for disease burden estimation. Here medical intelligence and the newly assembled MAP *Pf*PR database have been used to examine how existing spatial demographic databases can be used to improve PAR estimation. It is important to emphasise that these results are based on an opportunistic sample of the malaria endemic world.

### Low population densities

The mapping of areas of low population density has been used in the past to create masks of zero malaria transmission [15-17]. Results here suggest that contemporary spatial population databases are of insufficient detail to achieve this effectively. Figure 5 shows that, even using the global population database with the greatest number of input census units (GRUMP) and a zero population density threshold, 37 *Pf*PR surveys are excluded, with the majority of malaria surveys exhibiting prevalences above zero. As this threshold is increased, the number of surveys excluded rises. Moreover, no significant differences in *Pf*PR values were found at any of these thresholds between those surveys in the excluded areas and those surrounding them. Low population definitions cannot, therefore, be used to identify zero risk in malaria endemicity maps. Similar results were also found for the gazetted areas and are provided in additional file 1: Gazetted Areas.

Figure 2 demonstrates why the masking of epidemiologically relevant low population density areas is problematic. GRUMP incorporates the majority of the most contemporary and highest resolution spatially-referenced census data available, but for much of the malaria endemic world, this data is still many years old (Figure 2(b)) and at an administrative boundary level too low to identify small settlements (Figure 2(c)). Some of these problems may be alleviated with the release of GRUMP *beta *in late 2008, which will incorporate more contemporary, higher resolution data, alongside more recent UN country estimates and an improved water body mask (D. Balk, G. Yetman, pers. comm.). Steps have also been initiated to improve upon GRUMP using novel satellite sensor imagery-based approaches combined with detailed land cover information [28,49].

### High population densities and urbanization

Recent work has used urban extent maps to reduce classified risks in large urban areas for malaria mapping and PAR estimation [15,16]. Since this could only be implemented categorically in historical malaria maps, where endemicity values were binned into classes [17,55], this often resulted in the zeroing of risk. Table 3, however, suggests that low levels of transmission occur in the majority of urban areas for which *Pf*PR surveys were conducted. It is often not clear that all patients examined during the malaria survey have had their travel history checked and, thus, whether the sample is a true reflection of local transmission. Nevertheless, the results in Table 3 emphasise that urban areas, however they are mapped, should not be used as an absolute exclusion of malaria transmission. The exceptions to this rule are urban areas for which reliable ancillary data exist to confirm zero transmission. Such datasets, in the form of ITHGs, provide useful and explicit information on malaria free cities (Table 2), which can be linked to urban maps. Moreover, in combination with multi-year annual parasite index data and conservative exclusion masks based on measures of aridity and length of sporogony, these information have been used recently to produce maps of the limits of malaria transmission in 2007 [18,19].

Table 4 and Figure 6 provide evidence corroborating previous findings that urban areas suffer significantly reduced *P. falciparum *malaria transmission [13]. While there were insufficient sets of author-defined urban-rural *Pf*PR pairs to assess whether reduced transmission occurred in urban areas across Asia and the Americas, in Africa+ the differences were highly significant. It should also be emphasised that heterogeneity existed amongst the results, with 50 out of 273 urban-rural *Pf*PR pairs showing higher rates in areas mapped as urban, than those nearby mapped as rural. The results also followed the wet/dry climate-related patterns in malaria urban-rural transmission differences found by Hay *et al *[13]. For Africa+, Figure 6 reveals clear differences in the urban-rural *Pf*PR differential between NDVI zones, where those surveys conducted in wetter areas showed a clearer separation between urban and rural *Pf*PRs. The ratio between the medians remained the same between zones, however, with the median *Pf*PR in urban areas being around 40% of that in rural areas. In high transmission areas, where, for example rural *Pf*PR = 60%, this equates to a significant drop in entomological inoculation rate (EIR) from around 20 to 1 between rural and urban areas, respectively [56]. In lower transmission areas, this change is less substantial due to the non-linear PR-EIR relationship [56], with an EIR difference between rural and urban areas of just 0.9, for areas where rural *Pf*PR = 20%. These results are consistent with previous findings on the effects of urbanization on EIR, with transmission reduction being significantly larger in high transmission areas [13].

It is clear from Tables 5 and 6 that only the urban mask used to create GRUMP is of sufficient detail to identify those urban areas which have a significant effect on malaria transmission. Moreover, for those cities where sufficient *Pf*PR surveys exist within and near mapped extents, there are again clear differences in prevalence (Figure 7). The over-estimation of large urban extents exists within GRUMP-UE due to the 'over-glow' effect present in the night-time lights imagery used to produce it [29,48]. This effect means that the mapped extent of many well-electrified large settlements may also include less intensely-urban areas at their periphery, where malaria transmission may be significantly higher than that in the centre. Given the lack of globally consistent data on urban 'intensities', results here have shown that population density measures within urban extents can be used to discriminate between malaria-relevant urban and 'peri-urban' areas, identifying significant gradations that distinguish different levels of transmission (Figure 8).

Various recent studies have adjusted for urbanization in estimating malaria risk, PAR and burden over continental to global scales [13,15-17,57-60], but few have attempted to make use of medical intelligence information and assess the accuracy of the urban maps used, the epidemiological relevance of the urban-rural division adopted and the possibility of incorporating gradations of urbanization into these stratifications. This study highlights several ways that these data and relationships can be used in the mapping of *Pf *malaria endemicity. First, the ITHGs and other medical intelligence provide a valuable first step in identifying malaria free urban areas [18,19]. Second, when wanting to assign the urban/rural status for a malaria survey for which such information was not reported, the GRUMP-UE surface should be used. Third, incorporation of GPW population densities also enables discrimination of an epidemiologically relevant peri-urban class for survey assignment. Fourth, the significant relationships shown in Figure 7 indicate that, in the Africa+ region, rural, peri-urban and urban regions should be treated separately when the aim is to produce continuous prevalence or endemicity class predictions. Outside of Africa there is insufficient evidence from malaria surveys to support an informed decision.

Increasing computer power, the proliferation of geographical information systems (GIS) and the widespread availability of spatially-referenced human population census data have enabled significant advances in global spatial demography in the last decade [61], facilitating demographic denominators of disease risk to be estimated at sub-national scales, matching the spatial fidelity of advanced disease risk maps themselves. Such advances potentially facilitate the incorporation of sub-national spatial variations and burden estimates into major global disease burden assessments for the first time [62]. Recent trends are increasingly towards the use of spatial databases of human population and urbanization to estimate populations at risk, burdens, urbanization effects and infectious disease spread, e.g. [19,63-68]. Nevertheless, while emphasis is being placed more than ever upon mapping and incorporating spatial uncertainty into disease risk modelling, the uncertainties inherent in the estimates of the demographic denominators are often overlooked. Figures 2(b) and 2(c) highlight that, even in GRUMP, the population database with the most contemporary and highest resolution census data, much of the input data used is well over 10 years old and at low administrative unit level. The spatial and temporal errors and uncertainties that inevitably arise upon using these data for estimating present populations at risk at high resolution have yet to be quantified. Future work will examine approaches for estimating these uncertainties inherent in gridded population databases, to enable complete assessments of uncertainty in disease burden estimation. Moreover, the high resolution population mapping work will be extended beyond East Africa [28] to increase spatial detail and accuracies.

## Competing interests

The authors declare that they have no competing interests.

## Authors' contributions

AJT conceived, designed and implemented the research and wrote the paper. SIH aided with ideas, methodological and editorial input. CAG, CWK and AMN provided support in data compilation. The final version of the manuscript was seen and approved by all authors.

## Supplementary Material

Additional file 1 Gazetted Areas. Description and analyses based on gazetted areas data. Click here for file
