# Supplementary material for: Human population, urban settlement patterns and their impact on Plasmodium falciparum malaria endemicity
Source: Malar J. 2008 Oct 27;7:218. doi: 10.1186/1475-2875-7-218 (PMC2586635; doi:10.1186/1475-2875-7-218)
Supplement: Additional file 1 — Gazetted Areas. Description and analyses based on gazetted areas data. [file 1475-2875-7-218-S1.pdf]

Gazetted areas

The World Database on Protected Areas (WDPA) National sites dataset was downloaded from http://www.unep-wcmc.org/wdpa/. The dataset maps protected areas across the World into six categories based on primary management objectives, and each are described in detail on the WDPA website. In brief, the categories are as follows, I: Strict nature reserve or wilderness area, II: National park, III: Natural monument, IV: Habitat/species management area, V: Protected landscape, VI: Managed resource protected area. For each category and each NDVI class, the total area covered by the category in question was calculated, as well as the total population numbers and details of *Pf*PR surveys included. These data were plotted to visualise the effects of the different gazetted area categories on identifying areas of zero or low population density and *Pf*PR. For each category in each NDVI class, the mean *Pf*PR of the surveys excluded was compared to those remaining using a Mann-Whitney U-test to determine if the IUCN categories identified surveys with significantly lower *Pf*PRs.

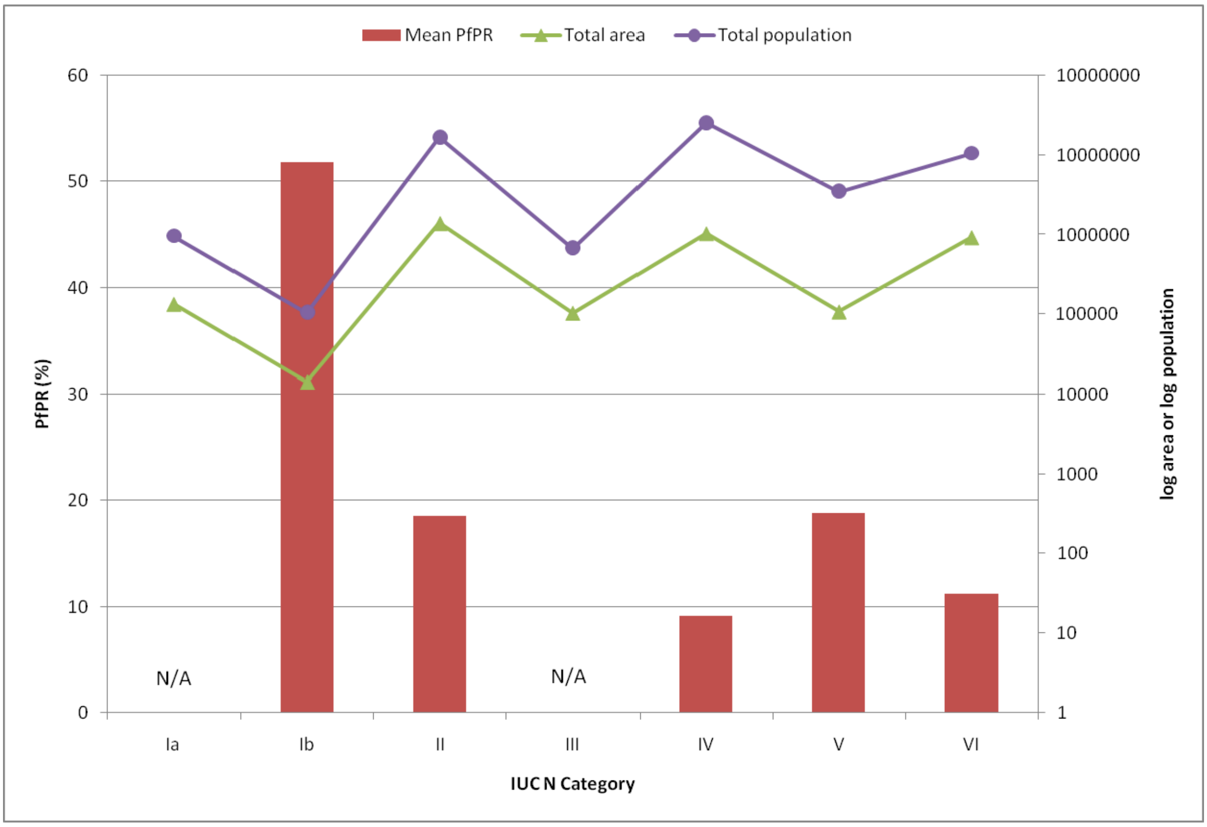

Figure 1. Population total, land area and number of PfPR surveys excluded by gazetted area categories.

Figure 1 demonstrates that, for the five IUCN gazetted area categories that contained *Pf*PR surveys, the mean *Pf*PR was well above zero. Moreover, large numbers of people reside within each the borders of each category, overall, indicating that such gazetted area classifications

cannot be used to identify areas of zero or low population density. Where sufficient surveys existed, no significant differences in *Pf*PR were observed between those surveys within each IUCN category of gazetted area and the remainder of the surveys.
